# How the microbiome challenges our concept of self

**DOI:** 10.1371/journal.pbio.2005358

**Published:** 2018-02-09

**Authors:** Tobias Rees, Thomas Bosch, Angela E. Douglas

**Affiliations:** 1 Social Studies of Medicine, McGill University, Montreal, Quebec, Canada; 2 Zoological Institute and Interdisciplinary Research Center Kiel Life Science, University of Kiel, Kiel, Germany; 3 Department of Entomology & Department of Molecular Biology and Genetics, Cornell University, Ithaca, New York, United States of America

## Abstract

Today, the three classical biological explanations of the individual self––the immune system, the brain, the genome––are being challenged by the new field of microbiome research. Evidence shows that our resident microbes orchestrate the adaptive immune system, influence the brain, and contribute more gene functions than our own genome. The realization that humans are not individual, discrete entities but rather the outcome of ever-changing interactions with microorganisms has consequences beyond the biological disciplines. In particular, it calls into question the assumption that distinctive human traits set us apart from all other animals––and therefore also the traditional disciplinary divisions between the arts and the sciences.

## Challenges to the classical biological explanations of the individual self

Are we humans really the individual, bounded selves we take ourselves to be? Until recently, little seemed more obvious to both the natural and the human sciences. The former found the material basis of the individual human self in the adaptive immune system, the brain, and the genome sequence, and the latter catalogued the many different ways in which humans—across time and space—have learned to make sense of what it means to be an individual self. The discrete self was a philosophical certainty in both the natural and the human sciences.

Today, this philosophical certainty––and therefore our sense of self––faces major challenges [1–4]. The source of this challenge is at first sight improbable: the study of microorganisms. It has been known from the inception of microbiology as a discipline in the 19th century (and arguably back to the invention of the microscope by van Leeuwenhoek in the 17th century) that animals, including humans, bear many microorganisms. Until recently, however, these microorganisms were generally treated as either pathogens or as insignificant: the absence of microbes was equated with health. This classical understanding of microbes has been called into question with the recent emergence of low-cost, high-throughput gene sequencing techniques that have enabled the study of microbial communities without cultivation. There is now overwhelming evidence that normal development as well as the maintenance of the organism depend on the microorganisms (collectively the microbiome [5]) that we harbor. The human is not a unitary entity but a dynamic and interactive community of human cells and microbial cells. By current estimates, approximately half of the cells in our body are microbial [6].

Life scientists, including clinicians, are increasingly recognizing the critical importance of the microbiome for what was previously regarded as self-enclosed human biology [7]. Microbiome science is leading to a major reassessment of biological processes as varied as the physiological function of specific organs, the composition of metabolites in body fluids, and the management of transmissible diseases. As microbiome research matures, broad patterns are emerging.

A first observation is that the key services that animals, from sponges to humans, gain from their microbiome are nutritional and defense against natural enemies. This commonality is unsurprising because interactions with the microbiome are near universal in extant animals and more ancient than the evolutionary origin of animals [8]: our ancestors were multi-organismal before they were multicellular.

**Fig 1:**
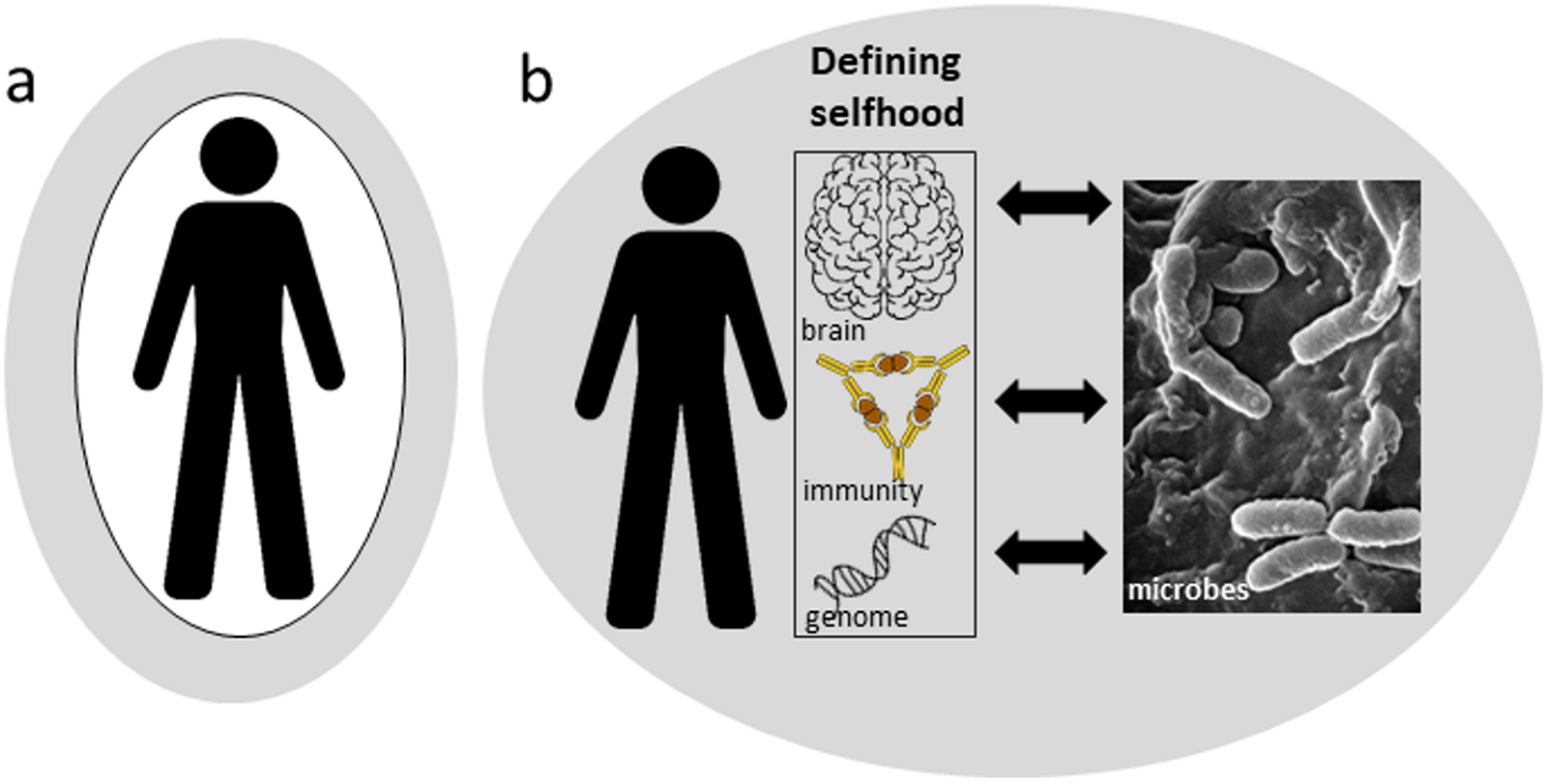
The changing perspective of the human self. (a) The traditional view: humans are set apart from nature. (b) View in the era of the microbiome: interactions with microorganisms define the individual human self.

A second, more surprising and challenging finding is that the microbiome also plays a central role in the three processes that have traditionally been said to define the human self: the adaptive immune system of vertebrates that discriminates self from nonself with exquisite molecular precision, the brain functions that underpin human personality and cognition, and the sequence of each person’s genome that guides our unique phenotypic traits (Fig 1).

Let us start with the adaptive immunity, which is a molecular recognition system generated by combinatorial rearrangement of gene segments. This recognition system is unique to each organism because the genetic changes are strictly somatic and the potential sequence diversity far exceeds the number of sequences that can be represented in the cell populations of an individual animal; in most vertebrates, including humans, the somatic recombination events are restricted to immunoglobulin genes in the progenitor cells of the two lymphocyte lineages (T cells and B cells). The microbiome does not influence these genetic events––but it plays a major role in shaping the abundance and activities of different types of T cells and B cells [9]. For example, the presence and composition of the gut microbiome can determine the profile of CD4^+^ T-cell populations in the intestine, inducing specific subsets of T_reg_ cells with anti-inflammatory functions and suppressing proinflammatory T helper 17 (Th17) and T helper 1 (Th1) cells [10,11]. In addition, short chain fatty acids, which are metabolic waste products of gut microorganisms, promote the differentiation of B cells into plasma cells that secrete protective immunoglobulin A molecules (IgAs) [12], and there is evidence that the gut microbiome has an important systemic effect of inhibiting the IgEs that mediate many allergic diseases (asthma, eczema, etc.), severe systemic reactions to allergens, and some autoimmune diseases [13,14]. Although many aspects of the complex crosstalk between the microbiome and adaptive immune system are not fully understood, it is already abundantly clear that the microbiome is part of the process that defines both whether the organism recognizes a specific molecular pattern as nonself and also how vigorously the organism responds to nonself. Immunologically speaking, self is not a human trait but the product of complex interactions between human cells and a multitude of microbial cells. Differently put, what has traditionally been called self is partly contingent on what has traditionally been called nonself.

Microbiome science is also confounding a long tradition in anatomy and physiology that defines our individual identity in terms of the higher functions of the human brain mediating self-awareness, personality traits, and emotional state [15]. It is perhaps disquieting for our understanding of our humanness and sense of self that the chief effects of the microbiome on nervous system function appear to relate precisely to these behavioral traits. Behavioral studies on rodents correlate perturbations or changes in the gut microbiome with cognitive function, social behavior, and stress-related responses akin to anxiety and depression; and anatomical and electrophysiological investigations are establishing a complex network of communication between products of gut microorganisms and central nervous system function [16]. Although at least some of the reported effects along the microbiota–gut–brain axis may be indirect (e.g., dependent on microbial effects on nutrition, metabolism, and immunity), research on rodent model systems is revealing that the microbiome is a major player in neurodevelopmental and neurodegenerative disease [17–19]. Therefore, the experimental evidence for microbiome effects on behavioral traits that we consider to define our sense of self––who and what we are––has profound implications beyond their biomedical significance and especially for our philosophical comprehension of the human self.

We are left with the third biological basis of self: the genome. The genome sequence of each human being is both fixed (barring somatic mutations) and unique (apart from identical twins). Superficially, the individuality of each human self is emphasized further by the microbiome, which is as unique to an individual “as its fingerprints” [20]. This microbial diversity is important because the microbiome associated with any one human contributes orders of magnitude more genes than the human genome, and microbial genes contribute to many phenotypic traits of the host, including nutritional and metabolic traits and the efficacy of therapeutic drugs [21–23]. The fact that important human traits cannot be defined exclusively by human genes would not disturb the genetic concept of self if the microbial partners and the genes they encode codiversified reliably with the human host. Although of foreign origin, the microbiome could be treated as “honorary self.” The problem, however, is that many aspects of the taxonomic and genetic composition of the microbial communities can vary independently of human genotype, both among individuals and over time within a single individual [24–26]. This fluidity in host–microbial relations has two profound consequences. The first––specific––one is that the scope of genome-based precision medicine [27] needs to be reassessed in the light of the evidence that many medically important traits are not shaped exclusively by the human genetic makeup but depend to a significant degree on the genetic capabilities of the microbiome [28]. The second––more far-reaching––consequence is that the growing realization that much of the genetic constitution of every human body is microbial [8] radically undermines any definition of “self” in terms of our individual human genome.

We want to stress that the discovery of the foundational importance of the microbiome for the genetic constitution of the human is qualitatively different from the much older argument that the environment has an influence over our genome. It is qualitatively different, first, insofar as the older distinction left untouched the assumption that it is our nuclear genome that is alone constitutive of the human individual self and merely granted the environment some “influence” over the genome. It is qualitatively different, second, because what is at stake is not actually influence: The microbiome is not “influencing” the genome; it is coconstituting the metaorganisms we humans are.

## Consequences and perspectives

How is the scientific endeavor responding to the growing realization that the composition and activities of our microbial partners are directly involved in the key biological processes that define traditional concepts of the self: from the responsiveness of our adaptive immune system to our cognitive capabilities and emotional states as well as the genetic basis of our individual phenotype and good health? To a large—but by no means universal—extent, the life sciences are coming to terms with the thought-provoking insights generated by the new discipline of microbiome science [7,29]. Indeed, we are witnessing sustained investments from academic, commercial, and funding organizations in microbiome research centers, funding initiatives, conferences, and colloquia. Textbooks are being revised and lecture courses redrafted to accommodate the new biology of the microbiome. This is not a time for business as usual in the life sciences.

**Fig 2:**
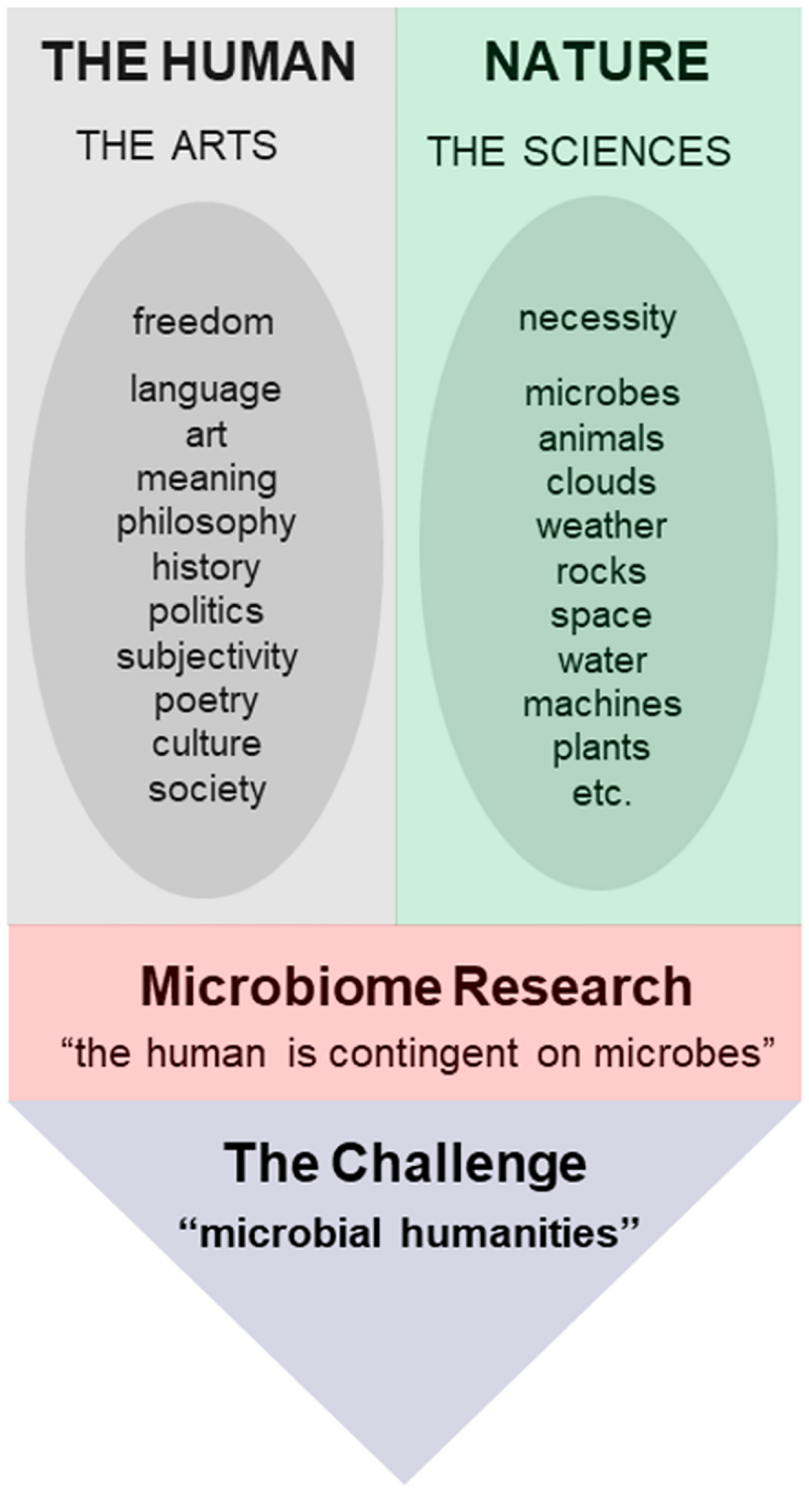
Microbiome research troubles the idea that the human is more than mere nature. A powerful dualism holds that humans are more than mere nature. A major consequence of this dualism is the emergence of two different kinds of sciences: the arts, concerned with manifestations of human freedom, and the sciences, studying nature as a realm of mechanical laws. Microbiome research troubles the idea that the human is more than mere nature because the human is contingent on microbes. How to render visible the human as a question in terms of the insight produced by microbiome research is a profound challenge of the contemporary, one that requires a radically new configuration of research beyond the arts and the sciences as they now exist.

A greater consequence is that the implications of microbiome science extend beyond the life sciences into the humanities (Fig 2). Indeed, as we see it, the finding that microorganisms are a constitutive part of ourselves calls for a new configuration of the effort to understand what it means to be human––to date the somewhat exclusive domain of the human sciences.

Historically, the division of labor between faculties of arts and faculties of science emerged in the 18th century, alongside the idea that humans are more than mere nature––that there are human-exclusive capacities that set us apart from “mere” animals and plants. More specifically, the argument was that reason, language, and art had liberated the human from the contingencies of nature and had gradually given rise to a uniquely human world, a world of “culture” that is irreducible to the laws of nature and that therefore requires its own set of sciences (the term “culture” was first used to mark a distinctive human world in the late 1770s). Arguably, the findings of microbiome research profoundly trouble the comprehension of the human that has sustained the traditional distinction between the natural sciences (concerned with the nonhuman) and the arts (concerned with the human as more than mere nature). Provocatively put, if humans depend on microorganisms, then what is at stake in the study of microbes qua microbes is not only an understanding of microorganisms but also the human. This doesn’t mean that the field of the arts can now be conveniently ploughed in terms of the natural sciences. On the contrary, it means that the stakes of the natural sciences exceed the expertise of the natural sciences and reach over into the arts. This makes a close collaboration of the life sciences with the human sciences imperative.

As we see it, it is important but not enough to argue that “we have never been individuals” [3]––or to suggest that human and microbial worlds are inseparably “entangled” [30–32]. What is needed, in addition, is a whole new configuration of research, one where arts and science are combined. The challenge is 2-fold. Researchers in the life sciences have to learn that the stakes of their research are bigger than their expertise, and researchers in the arts have to learn to think the human––philosophy, politics, and poetry––beyond the now untenable idea that humans are more than mere nature. The challenge is big, the opportunity even bigger: it is time, and perhaps past time, to rethink collaboratively––beyond arts and science divisions––what it means to be a living human being at home in a microbial world, one on which we depend and with which we are inseparably interwoven. Microbiome science has the exciting––the important––potential to catalyze the breakdown of the anachronistic barriers between the natural and the human sciences and enable a truly integrated understanding of what it means to be human, after the illusion of the bounded, individual self. The human is more than the human.

## Abbreviations

IgA: immunoglobulin A molecule
Th1: T helper 1
Th17: T helper 17
